# Effects of Moisture Content on the Acoustic Vibration and Modal Characteristics of Jinghu Soundboxes

**DOI:** 10.3390/ma19183853

**Published:** 2026-09-10

**Authors:** Yiyang Ge, Ying Li, Wen Fu, Rongzhen Song, Xiang Zhao, Shanyu Han, Zehui Jiang, Fuming Chen

**Affiliations:** 1 Institute of Biomaterials for Bamboo and Rattan Resources, International Centre for Bamboo and Rattan, Beijing 100102, China; 18226163254@163.com (Y.G.); 15074999173@163.com (Y.L.); wsw1394858791@163.com (W.F.); meizhen13581661026@163.com (R.S.); zhaoxiangyd@163.com (X.Z.); zehuijiang@icbr.ac.cn (Z.J.); 2 Key Laboratory of National Forestry and Grassland Administration, Beijing for Bamboo & Rattan Science and Technology, Beijing 100102, China; 3 School of Materials Science and Technology, Beijing Forestry University, Beijing 100083, China; 4 Jiangsu Co-Innovation Center of Efficient Processing and Utilization of Forest Resources, College of Materials Science and Engineering, Nanjing Forestry University, Nanjing 210037, China; hanshanyu@njfu.edu.cn

**Keywords:** bamboo moisture content, environmental humidity, acoustic vibration, modal analysis, estimated structural stiffness index

## Abstract

**Highlights:**

- Intermediate moisture levels enhance vibration responses of Jinghu bamboo soundboxes.
- Moisture reduces resonance frequency and estimated stiffness while increasing damping at extreme conditions.
- Humidity control is critical for maintaining the acoustic stability and structural durability of Jinghu soundboxes.

**Abstract:**

Bamboo is a renewable material widely used in traditional Chinese musical instruments, but its vibration behavior is sensitive to moisture. This study investigated how moisture content affects the vibration behavior of Jinghu bamboo soundboxes. Three Xipi and three Erhuang specimens were conditioned by soaking and drying to obtain 11 moisture states (M0–M10). Time domain responses, frequency response functions, natural frequencies, damping ratios, mode shapes, and a frequency-mass-based estimated structural stiffness index were evaluated using impact hammer testing and experimental modal analysis. Three individual Xipi soundboxes with nominal storage durations of 3, 46, and 86 years were examined before and after conditioning at 20 °C and 60% relative humidity as an exploratory case comparison. Both types showed stronger vibration responses at intermediate moisture levels, whereas nearly dry and nearly saturated conditions produced weaker responses. Increasing moisture generally lowered resonance frequencies and estimated structural stiffness index, while damping ratios were higher at the moisture extremes. Under the present test conditions, Xipi specimens exhibited a higher estimated structural stiffness index and stronger mid- to high-frequency responses than Erhuang specimens. Given the differences in specimen geometry and mass, these variations are interpreted as reflecting whole-structure dynamic responses rather than intrinsic differences in material properties. In the exploratory comparison, XP-86 showed the largest change, with a 61.17% decrease in fundamental frequency and the development of surface cracks. Given the limited number of independent specimens, the results are interpreted descriptively rather than as statistically generalizable effects. These results s demonstrate the importance of humidity control during the use and preservation of Jinghu soundboxes.

## 1. Introduction

The Jinghu is one of the most distinctive accompanying instruments in Peking Opera and is characterized by a bright, penetrating tone and strong sound projection [1]. Its bamboo soundbox is a key structural component involved in vibration transmission, energy dissipation, and sound radiation, with its density, elastic modulus, and damping properties influencing the resonance characteristics and vibration response of the instrument [2,3,4]. Bamboo has long been selected for Jinghu soundboxes based on traditional manufacturing experience [5]. Bamboo exhibits favorable vibration properties owing to its hierarchical and anisotropic structure, including the radial gradient distribution of vascular bundles, the fiber–parenchyma composite architecture, hierarchical porosity, and the viscoelastic cell-wall matrix composed primarily of cellulose, hemicellulose, and lignin [6,7,8,9,10]. Bamboo is also rapidly renewable and widely available, supporting its sustainable use in musical instrument manufacture [5,6].

Despite these advantages, bamboo, like wood, is hygroscopic and undergoes moisture adsorption and desorption in response to ambient conditions [11,12,13,14]. At a constant temperature and relative humidity, its moisture content tends toward an equilibrium moisture content (EMC). EMC is therefore an equilibrium state determined primarily by temperature, relative humidity, and sorption history rather than a fixed material constant. Changes in ambient temperature and relative humidity during performance, transportation, or storage may cause measurable shifts in the resonance and vibration response of Jinghu soundboxes, particularly when instruments are transferred between dry and humid climatic regions. Variations in ambient temperature and relative humidity alter the moisture content of bamboo, thereby affecting its mass, dimensions, mechanical properties, and vibration behavior. These effects may become particularly important when instruments are transferred between dry and humid climatic regions. Repeated moisture adsorption and desorption may additionally induce swelling and shrinkage, increasing the risk of deformation and cracking [12,13,14,15]. These effects complicate the manufacture, use, maintenance, and long-term preservation of Jinghu soundboxes and may compromise their vibration stability and structural durability [16,17,18,19,20,21].

From a structural perspective, water in bamboo can be distinguished as cell-wall-associated (bound) water and free water located mainly in cell lumina and larger voids. This distinction is important because the mechanical consequences of moisture depend strongly on whether the moisture content is below or above the fiber saturation point (FSP). Below the FSP, changes in moisture occur predominantly through adsorption or desorption at hydrophilic sites in the cell wall; these processes alter intermolecular hydrogen bonding, polymer-chain mobility, dimensional stability, stiffness, and viscoelastic energy dissipation [13,14,17,19,22,23,24,25]. Changes in cell-wall water below the FSP also cause swelling during adsorption and shrinkage during desorption. In bamboo, these dimensional changes are anisotropic and may vary radially because fibers and parenchyma differ in their structure and hygroscopic response. Rapid or repeated humidity variations may therefore generate non-uniform dimensional strains and increase the risk of deformation and cracking. Near and above the FSP, the cell wall is approximately saturated and further water uptake increasingly occurs as free water in lumina and pores. Additional free water contributes mainly to structural mass and fluid-related dissipation, while its incremental effect on cell-wall stiffness is expected to be smaller than that of bound-water changes. Therefore, moisture-induced changes in Jinghu soundboxes should not be interpreted as a single linear mechanism across the entire moisture range. Extremely low moisture may additionally increase brittleness and susceptibility to damage [26,27].

Although previous studies have examined the mechanical properties and macroscopic acoustic characteristics of bamboo, these aspects have generally been investigated separately [10,28,29]. As a result, the influence of moisture on bamboo resonant structures remains insufficiently understood, particularly for structures at the scale of musical instruments. Limited information is available on how a broad and controlled range of moisture conditions affects frequency response, damping, estimated stiffness, and modal behavior within the same soundbox structure. Long periods of storage and use may also be accompanied by changes in microstructure, mechanical properties, and moisture response [20,30,31]. However, individual Jinghu soundboxes with different storage histories have rarely been examined under the same controlled humidity conditions.

**Figure 1:**
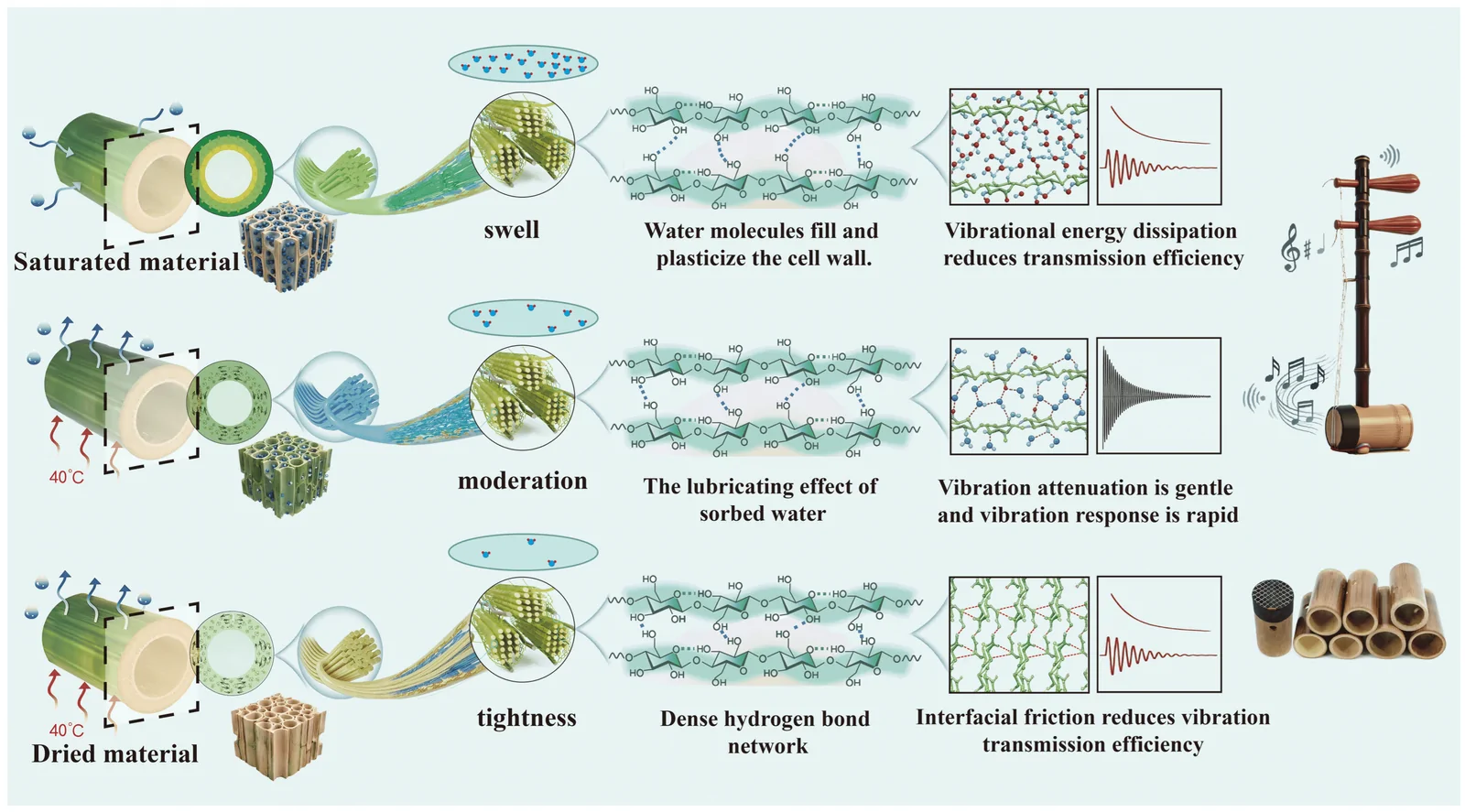
Influence mechanism of moisture content on the acoustic vibration behavior of Jinghu soundboxes.

Based on this background, this study investigated the influence of bamboo moisture content on the acoustic vibration characteristics of Jinghu soundboxes. Specifically, we hypothesized that increasing moisture content would generally reduce natural frequencies and the frequency-mass-based estimated structural stiffness index through added mass and cell-wall softening; that vibration amplitude would show a non-monotonic response, reaching higher levels at intermediate moisture contents than under near-dry or near-saturated conditions; and that damping would be elevated at the two moisture extremes because of dry-state interfacial losses and saturated-state water-related/viscoelastic dissipation. These relationships were expected to occur in both soundbox types, although absolute values could differ because of their distinct geometries and masses. First, Xipi and Erhuang soundbox specimens were evaluated over 11 moisture-content levels. Second, three individual Xipi soundboxes with nominal storage durations of 3, 46, and 86 years were examined before and after controlled-humidity treatment as an exploratory comparison of responses associated with different storage histories. By establishing various moisture conditions, we analyzed the dynamic vibration responses of the soundboxes and extracted key parameters, including natural frequencies, damping ratios, and mode shapes, using frequency response analysis and experimental modal analysis (Figure 1). The study aimed to quantify the relationships between moisture content and measurable vibration parameters, compare the responses of Xipi and Erhuang specimens, and discuss possible explanations in terms of changes in structural mass, stiffness, and energy dissipation.

## 2. Materials and Methods

### 2.1. Materials

Five-year-old moso bamboo (*Phyllostachys edulis* (Carrière) J. Houz.) was obtained from Fujian Province, China. All culms were harvested at the same time from the same bamboo stand. The specimens were prepared from the middle sections of different culms within the same harvested batch to reduce variation associated with harvest season and longitudinal position along the culm. The initial moisture content of the bamboo was 16.2%. Deionized water was used. Six bamboo soundboxes were cut according to Jinghu manufacturing standards, free from insect damage, discoloration, mold spots, and nodes, while preserving both the outer green and inner yellow layers. Inner and outer diameters were adjusted based on tuning requirements. The dimensions of the six bamboo soundboxes used for moisture regulation are shown in Table 1. Because Xipi and Erhuang soundboxes have different tuning requirements, their geometries were not identical. These dimensional differences were retained to represent the actual soundbox configurations used in practice. Accordingly, moisture-dependent changes were evaluated primarily within each specimen, whereas Xipi-Erhuang comparisons were interpreted as whole-structure responses that include the effects of geometry and mass.

For the exploratory comparison of soundboxes with different storage histories, three individual Xipi Jinghu soundboxes used for the dan role were selected. Their nominal storage durations, calculated relative to 2026, were approximately 86, 46, and 3 years, corresponding to manufacture in 1940, 1980, and 2023, respectively. The specimens were designated XP-86, XP-46, and XP-3. Their dimensional characteristics are presented in Table 2.

### 2.2. Moisture Content Regulation of Moso Bamboo Tubes

Six specimens (three Xipi and three Erhuang) were first soaked in deionized water at 18 °C for three days, then weighed to determine their initial moisture content, followed immediately by acoustic vibration performance testing. Subsequently, the specimens were placed in a 40 °C electrically heated constant-temperature drying oven. During the drying process, weights were recorded periodically along with acoustic vibration tests, ultimately generating 11 moisture gradient levels (M0–M10). M0–M10 are experimental moisture-content states along the imposed wetting/drying procedure. They are not 11 equilibrium moisture contents corresponding to 11 ambient climates. The results for each moisture state were summarized from the three independent specimens within each soundbox type. For each moisture condition, the reported vibration parameters represent the mean values calculated from the three specimens. Specific moisture gradient settings are detailed in Table 3. The moisture content at each test state was calculated on a dry-mass basis (absolute moisture content) according to

(1) $$M_{C}=\frac{{(m}_{i}-m_{0})}{m_{0}}\times 100\%$$

where *m_i_* is the measured mass at moisture state and *m*_0_ is the reference mass at the operational near-dry state M0. Because M0 was obtained using the 40 °C drying procedure described above, it is treated here as an experimental near-dry reference rather than as a standard oven-dry determination.

### 2.3. Controlled Climate Conditioning of Jinghu Soundboxes with Different Storage Durations

Owing to the limited availability of intact historical Jinghu soundboxes with traceable storage histories, only one specimen was available for each storage duration. Therefore, this experiment was designed as an exploratory case comparison rather than a statistical evaluation of storage-duration effects. To compare the responses of soundboxes with different storage histories under the same environmental conditions, the three specimens were placed simultaneously in a temperature and humidity-controlled chamber maintained at 20 °C and 60% relative humidity for more than two weeks. The specimens were weighed every 24 h to monitor changes during conditioning. When the mass change between two consecutive weightings was less than 0.002 g, the specimen was considered to have reached equilibrium. The specimens were then reweighed and subjected to acoustic vibration testing, allowing the changes in vibrational characteristics before and after exposure to the same controlled temperature and humidity environment to be compared. This comparison was not used to infer an age-dependent EMC.

### 2.4. Acoustic Vibration Response Testing

According to “Measurement of Sound Insulation in Buildings and Building Components” (GB/T 19889.3-2005) [32], the vibration response characteristics of six bamboo soundboxes at different moisture contents were tested using a hammer impact method with multi-point excitation, followed by modal analysis. The experiment employed a DASP instrument (V11, Institute of Noise and Vibration, China Oriental) for data acquisition and analysis. This system consists primarily of acquisition and analysis software, a dynamic signal analyzer (INV3062T), and sensors.

To ensure natural and smooth fitting of vibration mode shapes, sufficient numbers of measurement points must be evenly distributed across the specimen. Therefore, each bamboo soundbox was equipped with 20 measurement points, uniformly dividing the specimen into four equal sections, with point No. 8 designated as the reference point. During testing, an impact hammer excited the specimen sequentially from point 1 to point 20, with each point tested three times. The specimen was suspended using soft rubber bands at all four ends to simulate free boundary conditions and minimize the influence of constraints on vibration response. To improve signal quality, sound pressure sensors were placed away from nodal points of the vibration modes to ensure a high signal-to-noise ratio. The suspension, excitation, sensor arrangement, and data-acquisition procedure were kept consistent for all specimens and moisture conditions. The acquired time-domain signals were transformed into the frequency domain using a fast Fourier transform (FFT), and frequency response functions (FRFs) were obtained from the measured excitation and response signals. Modal parameters, including natural frequencies, damping ratios, and mode shapes, were identified from the FRFs using the PolyMAX frequency-domain modal identification method [33]. The resulting time- and frequency-domain data were subsequently used to determine the vibration parameters analyzed in this study.

When a material vibrates under excitation, the amplitude of the time-domain curve reflects the intensity of its vibration response, while the root mean square (RMS) value of the amplitude characterizes the level of vibration energy generated by the material system under excitation [34,35]. Under identical excitation conditions, a lower RMS indicates weaker vibration response energy, implying stronger vibration-damping performance of the material. According to Parseval’s theorem [35], the calculation is performed using Equation (2):

(2) $$\mathrm{RMS}\;=\sqrt{\frac{1}{n}\sum_{i=1}^{n}x_{i}^{2}}$$

where $x_{i}$ is the response value (Pa) at the pickup point.

### 2.5. Modal Analysis and Parameter Identification

Modal analysis reveals the inherent dynamic behavior of materials under various modes, which can be obtained through either computational methods or experimental testing. Each mode has a specific natural frequency, damping ratio, and mode shape. In this experiment, modal tests were conducted on bamboo tubes in a free state using a combined method of point by point excitation and multi-point response measurement, in accordance with the current national standard “Damping Materials, Test Method for Damping Performance” (GB/T 18258-2000) [36]. After obtaining modal parameters such as frequency, damping, and mode shapes from acoustic vibration characteristic tests, further modal identification was performed to determine the first three natural frequencies, modal damping ratios, and mode shapes. Because the natural frequency is affected by both material stiffness and specimen geometry, the frequency-based estimated structural stiffness index was used primarily to evaluate stiffness changes within specimens of the same geometry under different moisture conditions. For specimens with different geometries, the calculated values represent an estimated structural dynamic stiffness index reflecting whole-structure vibration behavior, rather than an intrinsic material stiffness. Based on the natural frequencies, the equivalent stiffness of the specimen was approximately calculated [37], expressed as

(3) $$K\;-\;\omega_{1}^{2}M=0$$

After rearranging Equation (3), we obtain

(4) $$\left|\frac{K_{1}}{K_{2}}\right|=\left|\frac{\omega_{1}^{2}M_{1}}{\omega_{2}^{2}M_{2}}\right|$$

where K is the equivalent stiffness, ω_1_ is the natural frequency, and M is the mass.

## 3. Results

### 3.1. Effects of Moisture Content on the Time-Domain Vibration Responses of Xipi and Erhuang Jinghu Soundboxes

**Figure 2:**
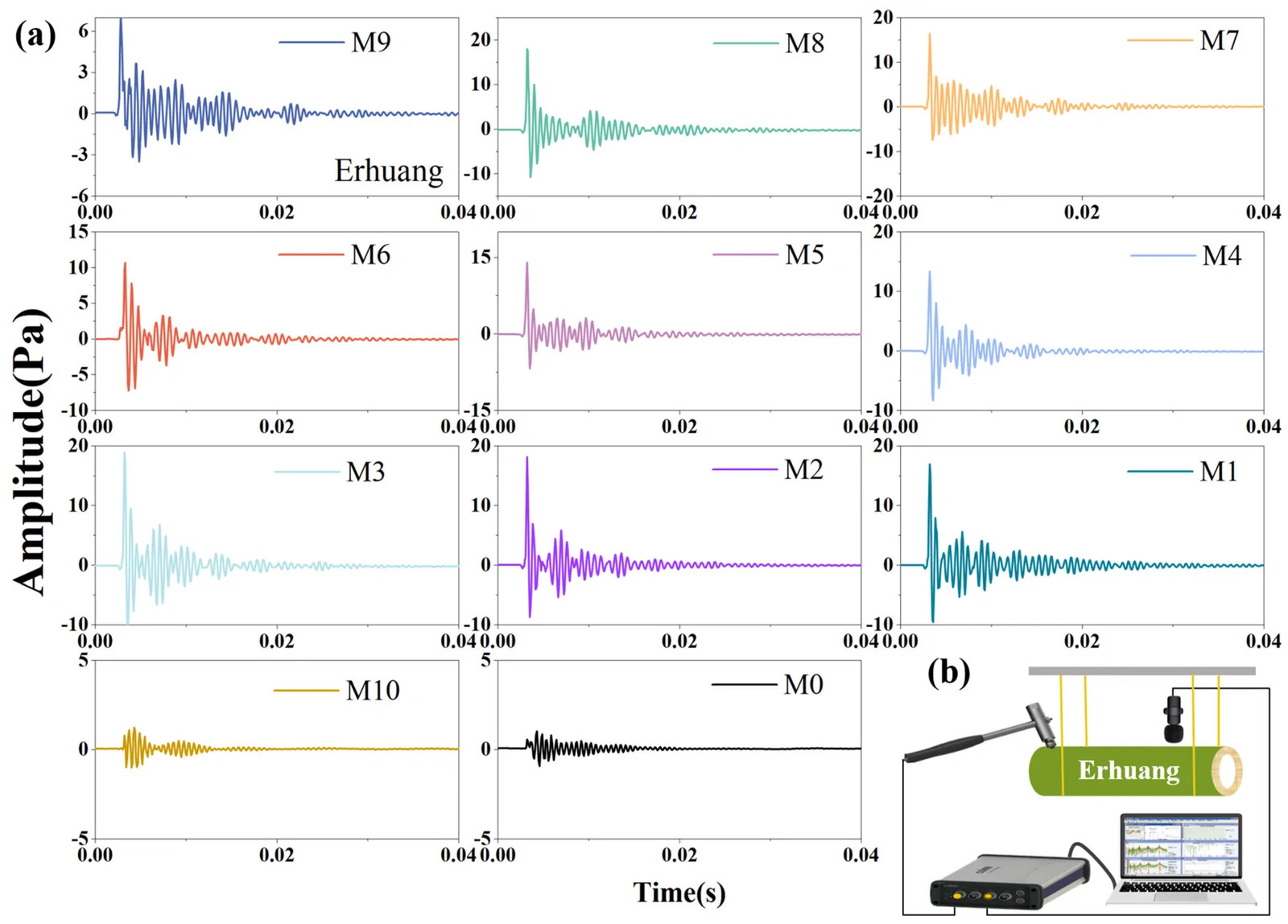
Acoustic vibration response signals and test setup of Erhuang bamboo resonance box. (**a**) Time-domain amplitude curves of acoustic vibration signals for different samples (M0–M10). (**b**) Schematic diagram of the acoustic vibration test system.

The acoustic vibration amplitude and damping of both Xipi and Erhuang resonance boxes exhibit systematic variations with changes in moisture content. Figure 2a shows the time-domain response curves of the Erhuang resonance box under different moisture levels (M0 to M10), while Figure 2b illustrates the corresponding excitation and measurement experimental setup. It can be observed that the initial vibration amplitudes of the bamboo tubes at M0 (completely dry) and M10 (fully saturated) are relatively low, and the vibrations decay rapidly. This indicates that a moderate moisture range (M1–M9) is more favorable for generating strong initial vibrations and maintaining effective sound radiation, whereas both completely dry (M0) and fully saturated (M10) conditions result in lower vibration excitation and energy output. The initial amplitudes of the bamboo tubes in the M1–M9 range are higher (6–20 Pa), decaying quickly within approximately 0.005 s and approaching zero by 0.03 s. As moisture content increases from M1 to M9, the vibration decay becomes progressively slower. In particular, during the M5–M9 stages, the vibration signals maintain noticeable amplitude fluctuations beyond 0.01 s, with an expanded fluctuation range (e.g., M9 exhibits fluctuations between −3.0 Pa and +3.0 Pa). Moisture-related changes in viscoelasticity and internal damping provide a plausible explanation. Overall, as moisture content increases from M1 to M9, the vibration decay slows down gradually, and the signal duration noticeably extends, reflecting a continuous modulation effect of moisture content on vibration characteristics: low moisture leads to rapid decay and high stiffness, while high moisture results in prolonged vibration and increased viscous damping. In the fully saturated state (M10), the cell cavities and micro-pores of the bamboo are filled with water, which absorbs vibrational energy, thereby greatly enhancing damping. Conversely, in the completely dry state (M0), the pores are empty and the cell walls are dense, resulting in weak damping but high structural rigidity [9], making it difficult to initiate vibration.

**Figure 3:**
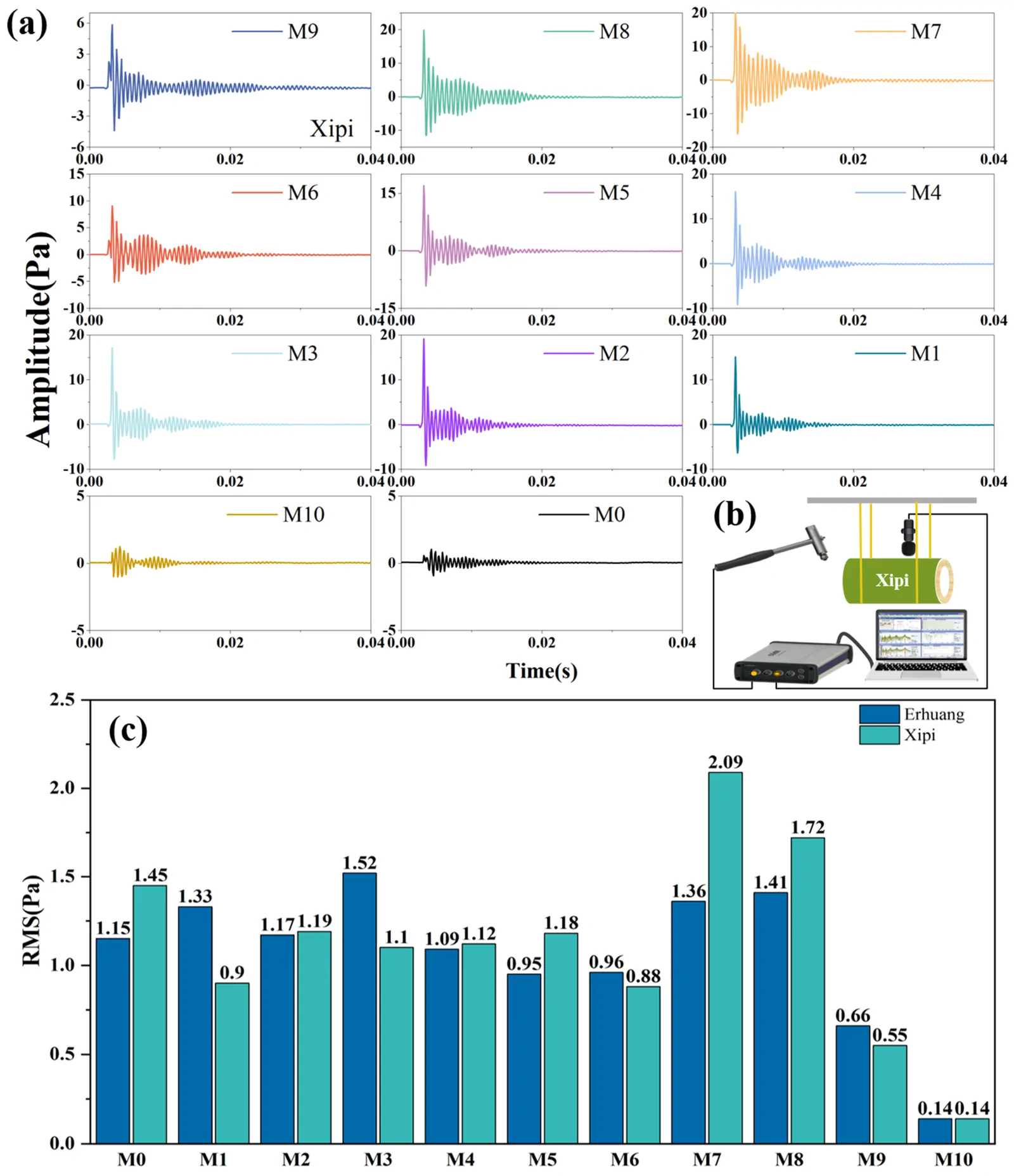
Acoustic vibration characteristics and test setup of Xipi bamboo resonance box. (**a**) Time-domain amplitude curves of acoustic vibration signals for different samples. (**b**) Schematic diagram of the acoustic vibration test system. (**c**) RMS amplitude comparison of acoustic vibration signals between Erhuang and Xipi samples.

Within a certain moisture range, the acoustic vibration performance of bamboo is optimal. However, when moisture content falls below or exceeds this range, vibration amplitude decreases and decay characteristics deteriorate, following a pattern of “poor performance at both extremes, best in the middle.” Figure 3 presents the variation of acoustic vibration characteristics of the Xipi resonance box with moisture content. Results show that the overall trends of time domain responses for Xipi and Erhuang resonance boxes are similar, yet marked differences exist under different moisture conditions (Figure 3a). Specifically, under completely dry (M0) and fully saturated (M10) conditions, the sound wave amplitudes remain low and decay rapidly, indicating high rates of internal energy dissipation in the bamboo. As moisture content increases gradually from M1 to M9, the vibration decay trend becomes increasingly gradual. From RMS analysis (Figure 3c), it is evident that, at the same moisture level, the RMS value of the Xipi resonance box is higher than that of the Erhuang. At M7 moisture content, the RMS value of Xipi reaches 2.09 Pa, compared to 1.36 Pa for Erhuang, a difference of about 53.7%. At M8 moisture content, the Erhuang achieves its peak RMS value of 1.41 Pa, still lower than Xipi’s 1.72 Pa under the same condition, approximately 18.0% lower. These results indicate that, under identical moisture conditions, the Erhuang resonance box exhibits lower vibration response and more pronounced damping behavior under the tested conditions. Regarding the trend analysis, in the low moisture range (M0–M1), RMS values increase with rising moisture content. Upon entering the medium moisture range (M2–M8), the Xipi maintains a relatively high RMS level, while the Erhuang shows a fluctuating upward trend. When moisture content enters the high range (M9–M10), RMS values of both types decrease markedly (at M10, both approach 0.14 Pa). Notably, the vibration peak of the Xipi bamboo soundbox occurs at moisture content M7, whereas that of the Erhuang is delayed to M8, with a more gradual sound pressure decay process. This difference reflects intrinsic distinctions between the two types of bamboo in terms of microstructure and moisture vibration coupling mechanisms [9,10,12].

### 3.2. Effects of Moisture Content on the Acoustic Vibration Characteristics of Xipi and Erhuang Jinghu Soundboxes

**Figure 4:**
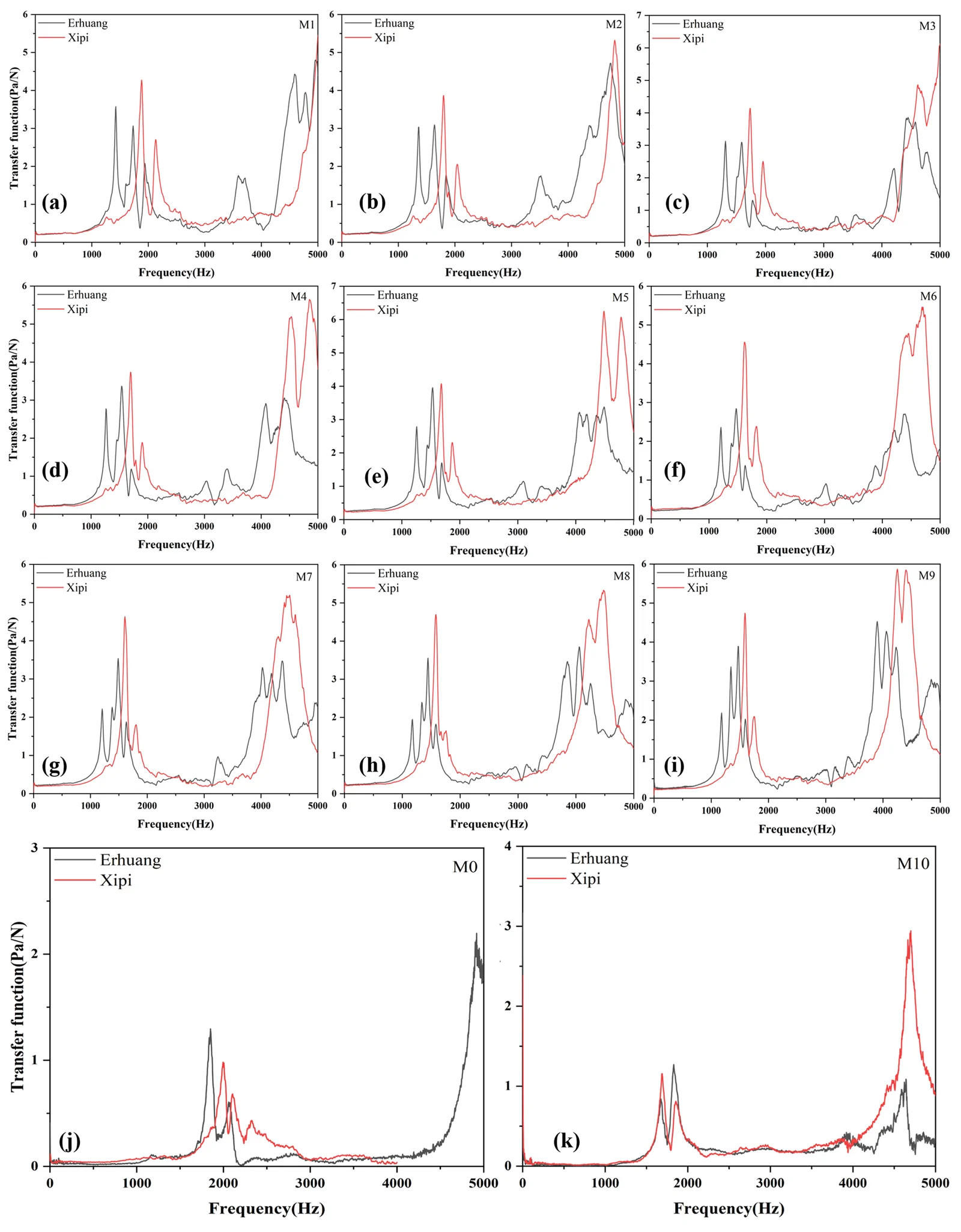
Frequency-domain transfer functions of Erhuang/Xipi bamboo resonance boxes under different moisture content gradients (M0–M10). (**a**–**k**) Transfer function curves corresponding to each moisture content (MC) group.

To compare the vibrational response differences between Xipi and Erhuang soundboxes under various moisture conditions, Figure 4a–k illustrates the frequency-domain transfer function variations for both Erhuang and Xipi soundboxes across different moisture gradients (M0 to M10). As moisture content increases from the completely dry state toward saturation, the resonance characteristics of both bamboo types exhibit distinct stage-wise evolution. In the low-moisture range (M0–M2), the transfer function curves are generally flat, with amplitudes mostly distributed between 1.0 and 6.0 Pa/N. The first three resonance peaks of Erhuang mainly concentrate within the 1000–2000 Hz frequency band, while the first two resonance peaks of Xipi are primarily centered around 2000 Hz, and the third peak extends toward approximately 5000 Hz. This indicates that the Xipi soundbox has higher structural rigidity, shifting its dominant energy transmission frequency range toward mid-to-high frequencies. As moisture content rises into the intermediate range (M3–M9), resonance peaks of both bamboo types further intensify and extend into the mid-to-high-frequency regions. Notably, although the amplitude of individual resonance peaks does not show a systematic upward trend, small fluctuations occur at different frequencies. This phenomenon may be related to uneven internal moisture distribution in the bamboo; such spatial variation in moisture can lead to non-uniform changes in local stiffness and damping properties, thereby affecting the consistency of vibration transmission. When the bamboo reaches full saturation (M10), the frequency response curve becomes overall flatter. Although a peak near 1500 Hz remains detectable, its amplitude decreases compared to earlier stages, and the response in the mid-to-high-frequency range is reduced. This result suggests that high moisture content reduces the overall stiffness of bamboo and increases system damping [1,9,10,38], causing substantial dissipation of vibrational energy in the mid-to-high-frequency bands. Comparing the two bamboo types, Xipi exhibits more pronounced responses in the mid-to-high-frequency range (3000–5000 Hz) than Erhuang, and its response fluctuates more prominently with changing moisture content. In contrast, in the low-to-mid-frequency range (1000–3000 Hz), Xipi demonstrates smoother and more continuous response characteristics. These performance differences arise not only from inherent differences in fiber arrangement density and cell wall structure but are also closely related to the penetration and distribution behavior of moisture within the bamboo. This further confirms that moisture content, as a key environmental factor, exerts a significant influence on the acoustic vibration performance of bamboo soundboxes, exhibiting strong material-structure dependency and gradient-dependent responses across moisture states [1].

**Figure 5:**
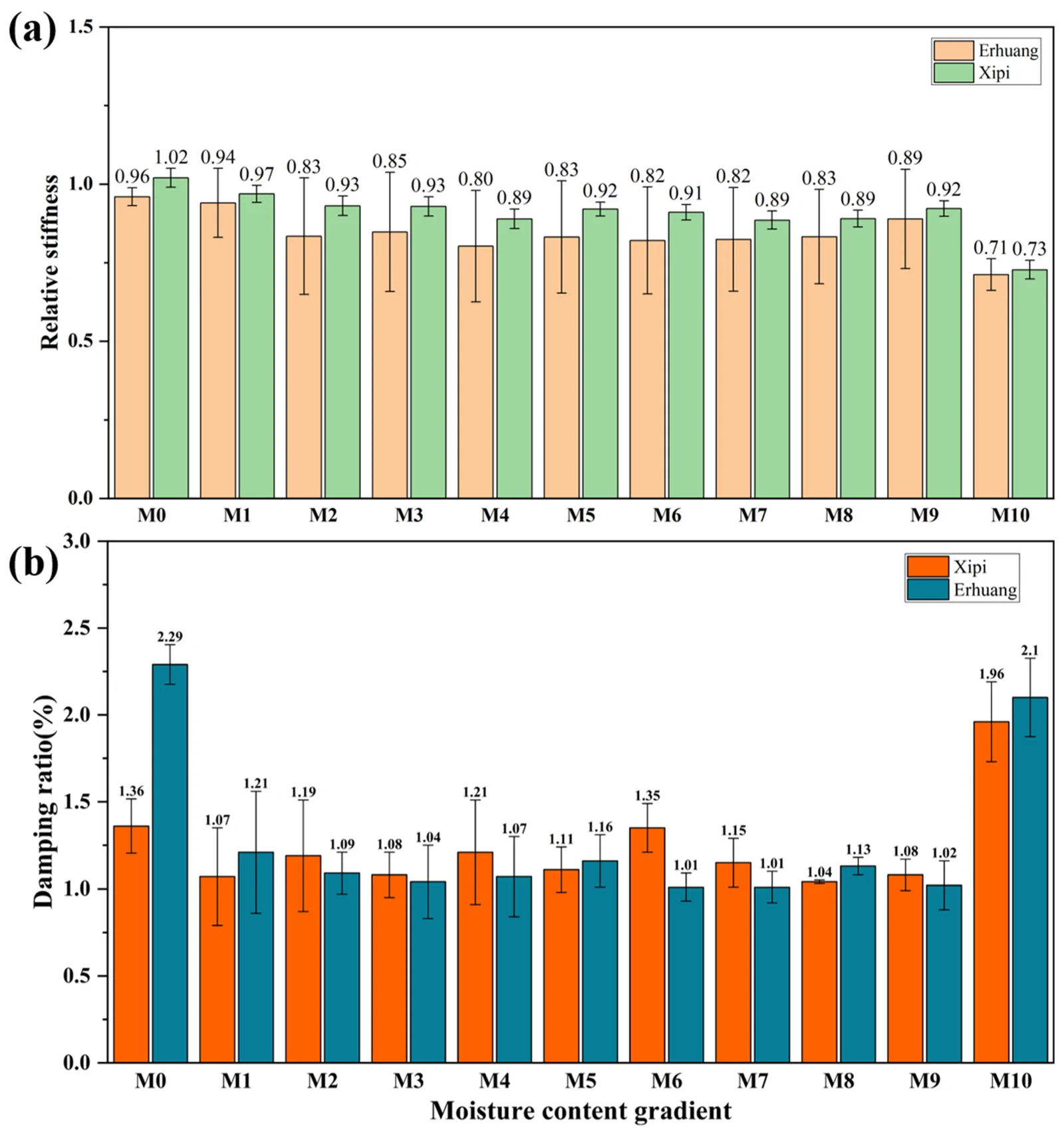
Estimated structural stiffness index and damping ratio of Erhuang/Xipi bamboo resonance boxes under different moisture content gradients. (**a**) Variation in estimated structural stiffness index with moisture content. (**b**) Variation in damping ratio with moisture content.

Moisture content modulates the dynamic characteristics and damping behavior of Erhuang and Xipi soundboxes through two mechanisms: stiffness reduction and internal energy dissipation. This is achieved by altering the chemical composition, brittleness, and viscoelastic properties of the bamboo cell walls. In the completely dry state, the cell walls are dense and brittle, and energy dissipation primarily results from microcrack propagation and interfacial friction. In the saturated state, water fills the microgaps within the cell walls, and energy dissipation shifts to mechanisms dominated by water-related losses and viscoelastic damping in the cell walls. These two distinct mechanisms together cause the bamboo soundboxes to exhibit relatively high damping ratios in both dry and saturated conditions, while their estimated structural stiffness index continuously decreases with increasing moisture content. Figure 5 shows the variation patterns of estimated structural stiffness index (a) and damping ratio (b) for Erhuang and Xipi soundboxes across different moisture gradients. In terms of estimated structural stiffness index (Figure 5a), its values of the Erhuang and Xipi soundboxes were 0.96 and 1.02, respectively, in the completely dry state (M0). When moisture content increased to the fully saturated state (M10), their estimated structural stiffness index decreased to 0.71 and 0.73, representing reductions of 26% and 28%, respectively. Overall, the Erhuang soundbox exhibited slightly lower estimated structural stiffness index than the Xipi soundbox under all moisture conditions, and both showed a monotonic decline in stiffness as moisture content increased. This indicates that lower moisture levels are more favorable for maintaining higher stiffness in bamboo tubes. Because the calculation incorporates frequency and mass and the specimen geometries differed, this between-type difference represents a whole-structure dynamic response rather than an intrinsic difference in bamboo stiffness. Due to its greater mass and relatively lower stiffness, the Erhuang soundbox has a lower natural frequency compared to the lighter and stiffer Xipi soundbox, which confirms from a dynamic perspective the experimental observation that the Xipi soundbox produces a higher pitch.

Regarding damping ratio (Figure 5b), the damping ratios of the Erhuang and Xipi soundboxes were 2.29% and 1.36%, respectively, in the completely dry state (M0). Within the moisture range from M1 to M9, both damping ratios decreased compared to the M0 condition; only when moisture content reached M10 did they rise again to 2.10% and 1.96%, respectively. Overall, at both M0 and M10 states, the Xipi soundbox had lower damping ratios than the Erhuang soundbox. However, between M1 and M9, the damping ratio of the Erhuang soundbox gradually approached that of the Xipi soundbox, with the difference progressively narrowing. Notably, although both dry and fully saturated bamboo exhibit high damping ratios, the underlying physical mechanisms differ fundamentally. In the dry state, the bamboo cell walls are dense and brittle, leading to significant internal energy dissipation due to microcrack propagation and enhanced friction at structural interfaces during vibration. In contrast, in the saturated state, water fills the cell cavities and microvoids, causing vibrational energy to be rapidly dissipated through water-induced energy loss and viscoelastic relaxation of softened cell walls [9,26,27]. These two mechanisms together explain why damping ratios remain high in both extreme moisture conditions.

### 3.3. Effects of Moisture Content on Acoustic Vibration Characteristics of Jinghu Soundboxes with Different Storage Durations

**Figure 6:**
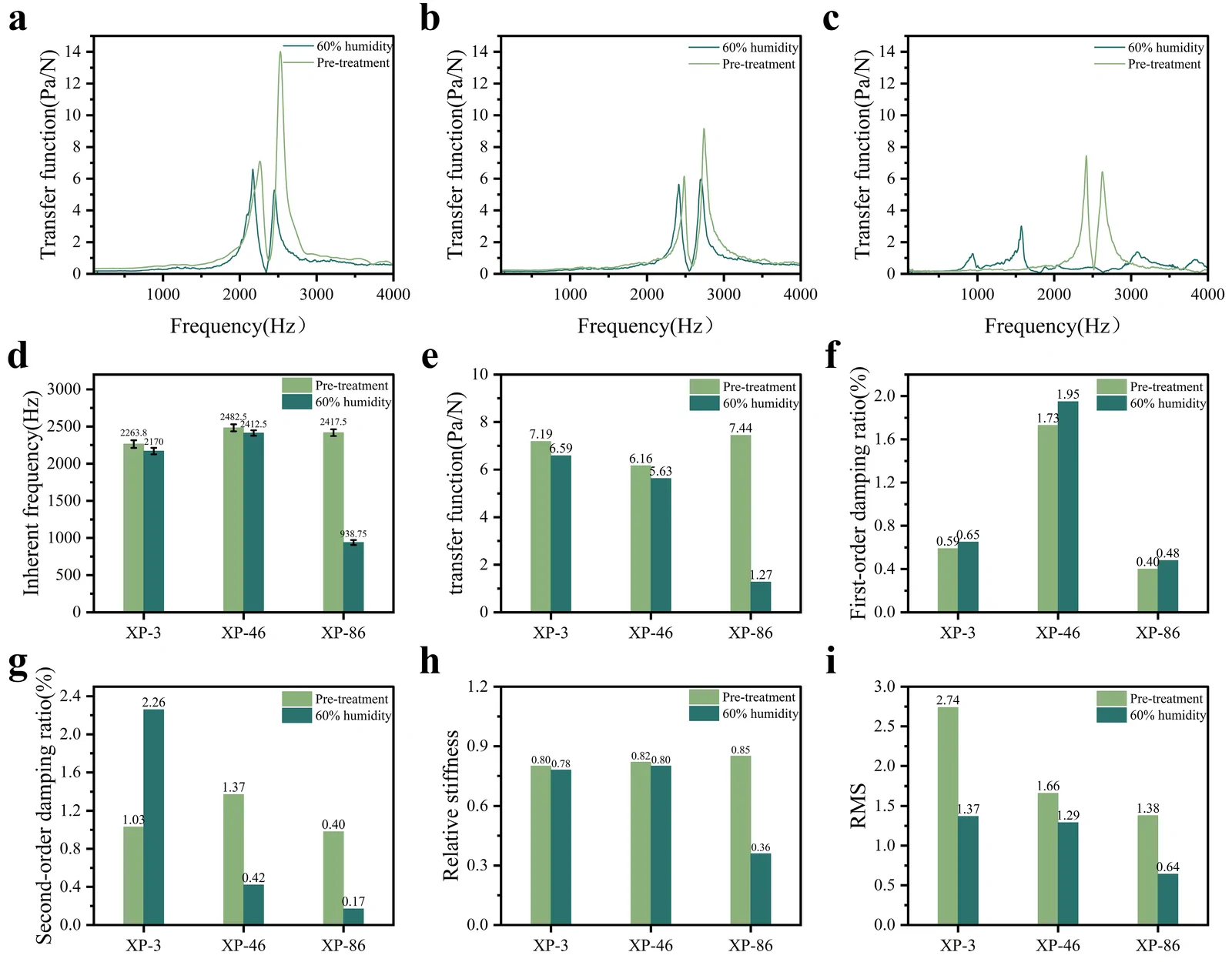
Effects of different ambient humidity on acoustic vibration characteristics of Jinghu bamboo tubes at three storage years. (**a**–**c**) Frequency response function curves of XP-86, XP-46 and XP-3. (**d**) Fundamental frequency of bamboo tube. (**e**) Frequency response function value corresponding to fundamental frequency of bamboo tube. (**f**,**g**) First- and second-order damping ratios of bamboo tube. (**h**) Estimated structural stiffness index of bamboo tube. (**i**) RMS value of bamboo tube.

Environmental humidity affects the acoustic vibration performance and surface quality of Jinghu soundboxes. Bamboo is a naturally hygroscopic material whose acoustic properties are highly sensitive to changes in environmental humidity [21]. After conditioning, XP-3, XP-46, and XP-86 gained 2.08 g, 2.11 g, and 2.33 g, respectively. These mass increases indicate moisture uptake as the three specimens approached a common state under the prescribed temperature and relative humidity conditions. However, because only one specimen was available for each storage duration, differences in mass gain cannot be attributed solely to storage age. Notably, surface cracks appeared on the XP-86 soundbox by day 5, showing that this specimen underwent structural damage during conditioning. Therefore, its subsequent vibration changes should be interpreted together with the effect of cracking rather than as a direct consequence of long-term storage. When bamboo absorbs moisture, water enters the cell-wall structure and causes dimensional swelling and changes in mechanical behavior [12,13,14,15,19]. As shown in the frequency response function curves (Figure 6a–c), conditioning under the common temperature and humidity environment was accompanied by shifts in the resonance characteristics of all three soundboxes. After humidity treatment, the XP-3 and XP-46 soundboxes still displayed two resonance peaks, whereas XP-86 developed a third resonance peak. Because XP-86 also developed visible surface cracks during conditioning, the additional resonance peak may be associated with a change in its structural condition; the present experiment does not allow this effect to be separated from possible differences related to storage history. The fundamental frequencies of the three Jinghu soundboxes with different storage durations were 2170 Hz, 2412.5 Hz, and 938.75 Hz, decreasing by 4.14%, 2.82%, and 61.17%, respectively, compared to humidity pre-conditions (Figure 6d). The corresponding transfer function values at the fundamental frequency also showed a declining trend (Figure 6e), measuring 6.59 Pa/N, 5.63 Pa/N, and 1.27 Pa/N, respectively. Among the three specimens, XP-86 showed by far the largest decrease in both fundamental frequency and transfer-function magnitude. Given the occurrence of surface cracking, this pronounced change is more appropriately interpreted as a combined response to moisture conditioning and structural damage rather than as evidence of an age-dependent effect. After humidity treatment, the first-order damping ratios of the three soundboxes all increased to some extent (Figure 6f). The second order damping ratio of XP-3 increased, whereas those of XP-46 and XP-86 decreased (Figure 6g). After moisture absorption, water entered the cell cavities and microgaps, which may enhance viscoelastic energy dissipation and thereby contribute to the observed increase in first-order damping [9,21,39]. However, the different second-order damping responses among XP-3, XP-46, and XP-86 cannot be attributed directly to storage duration because microfibril angle, molecular orientation, and other age-related microstructural parameters were not measured in this study. These differences should therefore be regarded as specimen-specific responses under the common conditioning treatment [31,40,41]. The frequency-based estimated structural stiffness index of XP-3 and XP-46 changed only slightly after conditioning (Figure 6h), whereas that of XP-86 decreased markedly from 0.85 to 0.36. Because XP-86 developed surface cracks during conditioning, the large decrease in this index is likely associated, at least in part, with the change in structural integrity. Figure 6i shows the root mean square (RMS) values of XP-3, XP-46, and XP-86, which were 1.37, 1.29, and 0.64, respectively, representing reductions of 50%, 22.29%, and 53.62% compared with their pre-conditioning values. Overall, the three specimens responded differently to the same controlled temperature and humidity environment, with XP-86 exhibiting the largest changes. Nevertheless, because only one specimen was tested for each storage duration and XP-86 experienced cracking, these results should be interpreted as an exploratory comparison of individual specimens rather than as evidence for a systematic storage-age effect.

### 3.4. The Influence of Moisture Content on the Vibration Modal Behavior of Jinghu Resonator Bodies with Different Storage Durations

**Figure 7:**
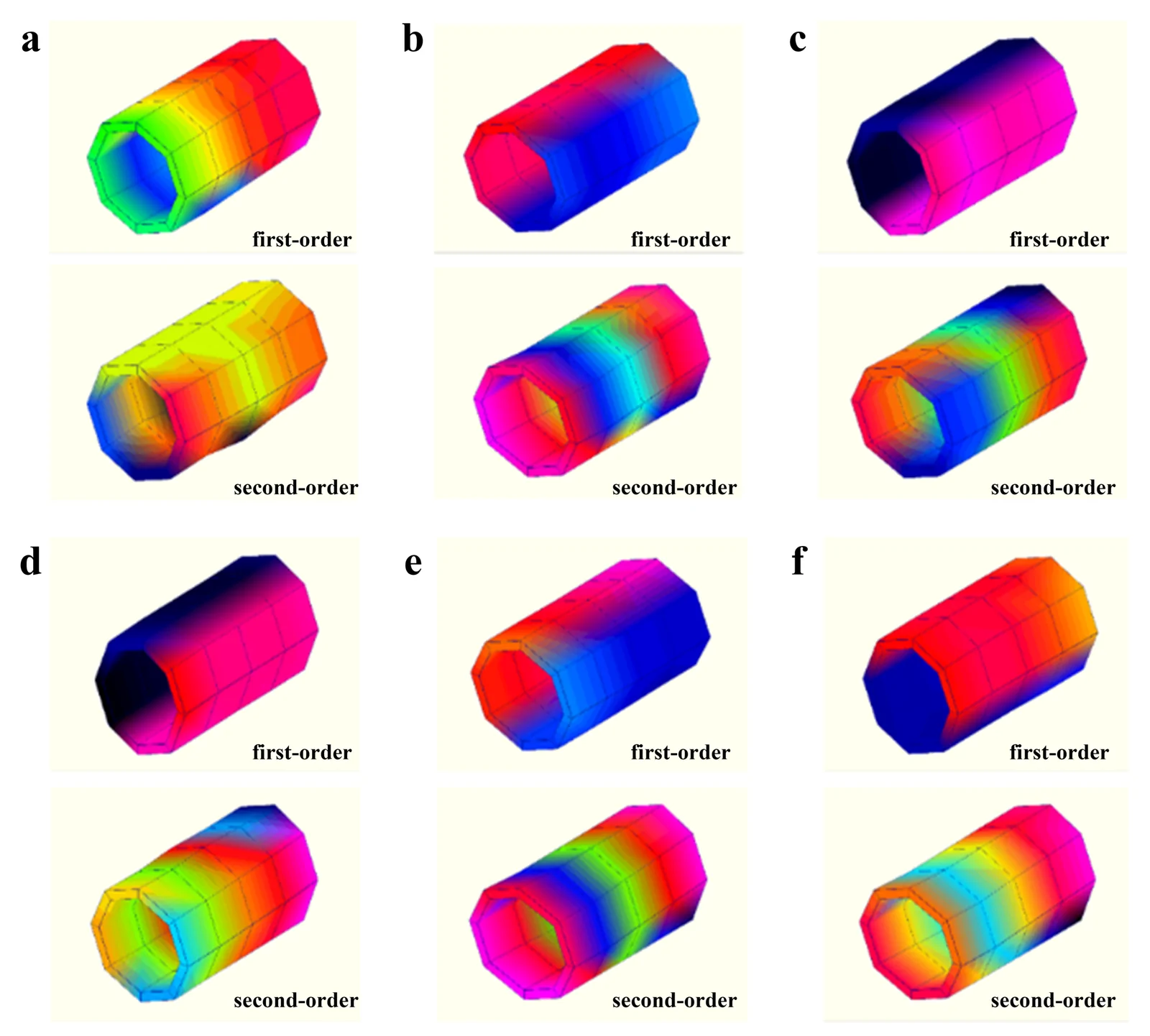
Modal shape changes of Jinghu soundboxes at three storage years under ambient humidity. (**a**–**c**) Mode shapes of XP-86, XP-46 and XP-3 after moisture conditioning. (**d**–**f**) Mode shapes of XP-86, XP-46 and XP-3 before moisture conditioning.

Environmental humidity directly affects the modal shapes, natural frequencies, and vibration response characteristics of Jinghu resonator bodies by altering the moisture state and structural properties of bamboo. In the present study, the three soundboxes with different storage histories showed different modal responses after exposure to the same controlled temperature and humidity conditions. However, because only one specimen was available for each storage duration, these differences should not be interpreted as evidence of a systematic coupling between storage duration and humidity response. After moisture conditioning (Figure 7a–c), the modal shapes of three resonator bodies, XP-86, XP-46, and XP-3, with different storage durations showed distinct characteristics, differing from their pre-conditioning states (Figure 7d–f). After conditioning, XP-86 exhibited an axial bending mode as its first order mode and a higher order bending combined with local coupled mode as its second order mode. Both XP-46 and XP-3 displayed axial bending as their first order mode and torsion bending coupled modes as their second order mode after conditioning, whereas prior to conditioning, both had relatively weaker coupling degrees and deformation amplitudes in their modal shapes. The change in modal shapes is fundamentally determined by alterations in the distribution of mass and stiffness within the bamboo material. As environmental humidity increases, water molecules enter the multi scale pores of bamboo through adsorption via hydrophilic groups on cell walls and capillary action, increasing the overall mass of the resonator body [24,42]. Simultaneously, when moisture penetrates the amorphous regions of the cell wall, hydrogen bonds between cellulose molecular chains are weakened, causing swelling of the amorphous cellulose and matrix, which leads to a reduction in cell wall stiffness [25]. This coordinated change in mass and stiffness directly alters the vibrational response patterns at resonance frequencies. In addition, XP-86 developed visible surface cracks during conditioning, which changed its structural integrity and may have contributed to the more pronounced local deformation observed in its second-order mode. Therefore, the modal behavior of XP-86 should be interpreted as a specimen-specific response involving both moisture conditioning and structural damage. XP-46 and XP-3 retained comparatively more regular torsion–bending coupled second-order modes after conditioning and did not show the visible cracking observed in XP-86. Nevertheless, these differences should be regarded as an exploratory comparison among individual soundboxes with different storage histories rather than as evidence that shorter storage duration preserves greater elasticity or structural integrity. Further studies using replicate specimens and direct characterization of age-related structural properties are required to distinguish the effects of moisture, structural damage, and long-term storage.

## 4. Conclusions

This study investigated the effects of moisture content on the vibration and modal characteristics of Xipi and Erhuang Jinghu bamboo soundboxes, together with an exploratory comparison of three Xipi soundboxes with different storage histories. Both soundbox types exhibited stronger vibration responses at intermediate moisture levels, whereas near-dry and near-saturated conditions resulted in reduced vibration responses. Increasing moisture content generally decreased resonance frequency and the frequency-mass-based estimated structural stiffness index, while damping ratios increased under extremely dry and saturated conditions. The observed moisture-dependent responses agree with previous studies on hygroscopic bamboo and wood materials, indicating that moisture influences acoustic performance through coupled changes in structural mass, stiffness, and energy dissipation. At the structural level of Jinghu soundboxes, moderate moisture conditions promoted vibration efficiency, whereas excessive moisture increased damping and reduced stiffness. Differences between Xipi and Erhuang soundboxes were observed in vibration amplitude and frequency response characteristics; however, these differences should be interpreted as whole-structure responses because of variations in geometry and mass. The exploratory comparison of soundboxes with different storage histories showed that XP-86 exhibited the greatest change after humidity conditioning, including a substantial decrease in fundamental frequency. However, the development of surface cracks during conditioning indicates that this response resulted from the combined effects of moisture exposure and structural damage rather than storage duration alone. The limited number of historical specimens and the absence of direct microstructural characterization represent important limitations of this study. Further investigations using larger specimen populations, controlled humidity cycling, and microstructural analyses are required to clarify the mechanisms governing moisture–vibration interactions in bamboo musical instruments. Overall, this study demonstrates that moisture content is a critical factor affecting the acoustic vibration stability of Jinghu bamboo soundboxes and provides experimental evidence supporting humidity control during their use, transportation, and preservation.

## Figures and Tables

**Table 1:** Dimensions of bamboo soundboxes.

Types	Mass/g	Outer Diameter/mm	Inner Diameter/mm	Length/mm	Density/(g/cm^3^)
Erhuang1	101.39	76.14	64.79	118.70	0.68
Erhuang2	104.91	71.50	59.47	123.16	0.69
Erhuang3	114.93	73.02	61.60	120.74	0.79
Xipi1	83.08	65.02	54.10	109.60	0.74
Xipi2	85.68	67.14	56.75	109.80	0.77
Xipi3	88.12	72.52	61.48	110.10	0.69

**Table 2:** Dimensional specifications of Jinghu soundboxes with different storage durations.

Types	Mass/g	Outer Diameter/mm	Inner Diameter/mm	Length/mm	Density/(g/cm^3^)
XP-86	93.13	57.70	45.60	120.58	0.68
XP-46	74.40	55.68	43.90	114.85	0.69
XP-3	76.61	60.80	48.35	116.70	0.69

**Table 3:** Moisture gradient levels of bamboo tubes.

Type	Moisture Content/%	Type	Moisture Content/%
M0	Approaching Dryness (0)	M6	33.95 ± 6.17
M1	6.80 ± 2.71	M7	40.73 ± 6.62
M2	10.98 ± 3.09	M8	46.38 ± 6.74
M3	17.62 ± 4.28	M9	50.73 ± 6.67
M4	25.41 ± 5.25	M10	Approaching Saturation (100)
M5	29.21 ± 5.86	—	—

## Data Availability

The original contributions presented in this study are included in the article. Further inquiries can be directed to the corresponding author.
